# Quantitative Profiling of Bile Acids in Feces of Humans and Rodents by Ultra-High-Performance Liquid Chromatography–Quadrupole Time-of-Flight Mass Spectrometry

**DOI:** 10.3390/metabo12070633

**Published:** 2022-07-11

**Authors:** Xiaoxu Zhang, Xiaoxue Liu, Jiufang Yang, Fazheng Ren, Yixuan Li

**Affiliations:** 1 Key Laboratory of Precision Nutrition and Food Quality, Department of Nutrition and Health, China Agricultural University, Beijing 100193, China; xiaoxuzhang0220@cau.edu.cn (X.Z.); renfazheng@263.net (F.R.); 2 College of Food Science and Nutritional Engineering, China Agricultural University, Beijing 100083, China; lxiaoxue0828@163.com; 3 Institut Necker Enfants Malades, Université Paris Cité, F-75015 Paris, France; jiufang.yang@inserm.fr

**Keywords:** bile acids, UPLC–Q-TOF, wet feces, sulfation, isomerization, BA indices

## Abstract

A simple, sensitive, and reliable quantification and identification method was developed and validated for simultaneous analysis of 58 bile acids (BAs) in human and rodent (mouse and rat) fecal samples. The method involves an extraction step with a 5% ammonium–ethanol aqueous solution; the BAs were quantified by high-resolution mass spectrometry (ultra-high-performance liquid chromatography coupled with quadrupole-time-of-flight mass spectrometry, UPLC–Q-TOF). The recoveries were 80.05–120.83%, with coefficient variations (CVs) of 0.01–9.82% for three biological species. The limits of detection (LODs) were in the range of 0.01–0.24 μg/kg, and the limits of quantification (LOQs) ranged from 0.03 to 0.81 μg/kg. In addition, the analytical method was used to identify and quantify BAs in end-stage renal disease (ESRD) patients, C57BL/6 mice, and Sprague-Dawley (SD) rats. The fecal BA profile and analysis of BA indices in these samples provide valuable information for further BA metabolic disorder research.

## 1. Introduction

Bile acids (BAs) are biosynthesized in hepatocytes from cholesterol, which present an important biological function in humans and animals, such as digestion and absorption of lipids and other fat-soluble components [1]. BAs synthesized in the liver are called primary BA (PBA). PBAs’ composition is quite different in different biological species. In humans, cholic acid (CA) and chenodeoxycholic acid (CDCA) are major PBAs; in addition, muricholic acid (MCA) is also a PBA in rodents PBAs are synthesized by the classical and alternative pathways. The classical pathway is initiated via 7α-hydroxylation of cholesterol under 7α-hydroxylase (CYP7A1) action and then 12-α hydroxylation of the intermediates by sterol 12-α hydroxylase (CYP8B1), followed by side-chain oxidation by sterol 27 hydroxylase (CYP27A1). The alternative pathway begins with the hydroxylation of the cholesterol side chain by CYP27A1, followed by 7-α hydroxylation of the oxysterol intermediates by oxysterol 7-α hydroxylase (CYP7B1). In rodents, most CDCA is immediately converted into MCA [2]. Then PBAs are amidated with glycine and taurine in the liver rapidly, then flow into the duodenum [1]. The conjugated BAs mainly incur deconjugation, 7α-dehydroxylation, oxidation, and epimerization reactions in the colon by several bacteria to produce secondary BAs (SBA) [3,4,5]. Other reactions also undergo liver and intestinal metabolisms, such as sulfation, glycosyl esterification, and glycosylation [6,7]. Almost 95% of the BAs are reabsorbed and recycled during the enterohepatic circulation, and only 5% are excreted in the feces [8].

Fecal BAs act as important biomarkers and signaling molecules in several studies because of the complex interplay between BAs and gut microbiota [9,10]. Gut microbiota could modify the BA pool composition and size, especially disordered gut microbiome producing abundant of conjugated BAs and BA structurally similar metabolites, which could indirectly indicate disease states [10,11,12]. In turn, some BAs might have antimicrobial activities, such as changing the pH of the intestinal microenvironment or disrupting intestinal microbial membranes [13]. Since BAs are regarded as a communication bridge between the gut microbiome and the various organs, such as liver and brain (gut–liver–brain axis), more and more studies focus on the fecal detection of BAs to find the relationship between diseases and intestinal microorganism [10,14,15,16,17]. More than that, BAs have a correlation with aging [12,18,19]. Sato et al. found some particular SBA, including iso-, 3-oxo-, allo-, 3-oxoallo-, and isoallo-lithocholic acid in centenarians’ feces, which generated by enriched gut microbes [13]. Based on these, developing a robust, accurate, and high throughput BA analysis method in feces is extremely important. However, the multiple variations of BA structure and their similar chemical properties have presented challenges in their separation and detection.

Different from the serum or plasma matrix, the fecal matrix is more complex because of the presence of proteins, lipids, salts, and others. These impurities lead to difficulty in fecal BAs detection. Moreover, the feces condition, wet or dry, also affects the extraction efficiency. Recently, Shafaei et al. compared the extraction efficiency of 12 BAs from wet and dry feces [8]. They found that the recoveries of all the target BAs were quite lower in dried fecal samples than in wet samples. Especially, the glycine conjugated BA recoveries were below 30%. Therefore, an appropriate BA preparation process is quite necessary for accurate qualitative and quantitative analysis, especially when LC-MS is used as the detector. The common preparation processes include BA extraction, purification, and dilution for high BA concentration. A small amount of ammonium hydroxide or sodium hydroxide was often added to the extraction solution to attenuate the binding of proteins to BAs, thus improving fecal BAs extraction efficiency [6]. Solid-phase extraction (SPE) and liquid–liquid extraction (LLE) are two optional purification and concentration methods [6,20]. An efficient preparation procedure could decrease matrix effects and improve sensitivity in BAs analysis. Therefore, for different experimental substrates, pre-treatment methods still need to be optimized.

Many studies have reported BA detection technologies in the last few decades, including thin-layer chromatography (TLC), high-performance liquid chromatography with UV detection (HPLC-UV), gas chromatography with flame ionization (GC-FID), gas chromatography mass spectrometry (GC-MS), liquid chromatography mass spectrometry (LC-MS), ultrahigh-performance liquid chromatography-tandem mass spectrometry (UHPLC-MS/MS), enzyme-linked immunosorbent assay (ELISA), and nuclear magnetic resonance spectroscopy (NMR) [20,21,22]. Recently, high-resolution mass spectrometers (HRMS), such as Orbitrap or TOF, have been increasingly used for BA identification, characterization, and quantifications. These HRMS offer high mass resolution (>100,000 fwhm) and high mass accuracy (<5 ppm). They could improve the BA isomers’ separation, sensitivity, and specificity [23,24]. Importantly, once full-scan mass spectra are obtained during sample acquisition, valuable information about other BA metabolites, metabolite modifications, or degradation products could be available for further data analysis.

Hence, our aim was to develop a simple, robust, and reliable bioanalytical method for wide structural coverage of BA analytes (Mono-OH, Di-OH, Tri-OH, and oxo-, nor-, iso-) measured quantitatively in feces from clinical and preclinical study samples (two rodents, rats and mouse) with UPLC–Q-TOF. Our research group was studying the abnormal lipid metabolism in end-stage renal disease patients (ESRD) who lost renal function. These patients are always accompanied by lipid metabolic disturbance and intestinal microbiota disorders. Thus, the feces of ESRD patients were chosen as the clinical sample to validate our BA method. The feces of rodents (C57BL/6 mice and Sprague- Dawley rats) were also used to validate the method. Finally, the BA indices were compared in these biological species, especially the SBA composition analysis, to provide basic data for future gut/intestinal-X axis research.

## 2. Result and Discussion

Along with the discovery of the important role of gut microbes in diseases and health, more and more studies have focused on the composition and changes of microbial metabolites, especially for BAs. The composition of microbes and the regulation of the micro-environment on microbial metabolism would change the structure of BAs, causing them to isomerize and form variable isomers, which play an important regulatory role in humans and animals. Many studies have developed and profiled the BAs in plasma and urine of multiple biological species, such as humans, monkeys, rabbits, rats, and mice [20,25,26,27,28]. However, it is crucial to profile various BAs in feces because the fecal BA could evaluate intestinal microorganism status directly [6,29,30]. Therefore, our goal was to develop a robust, high throughput method for comprehensive analysis of fecal BA in human and preclinical animals (mouse and rat).

### 2.1. BA Extraction Methods Comparison

Shafaei et al. found poor BA recovery present in dried fecal material, while wet samples could provide better efficiency and repeatability [8]. Hence, our extraction approach optimization was carried out on wet feces.

**Figure 1:**
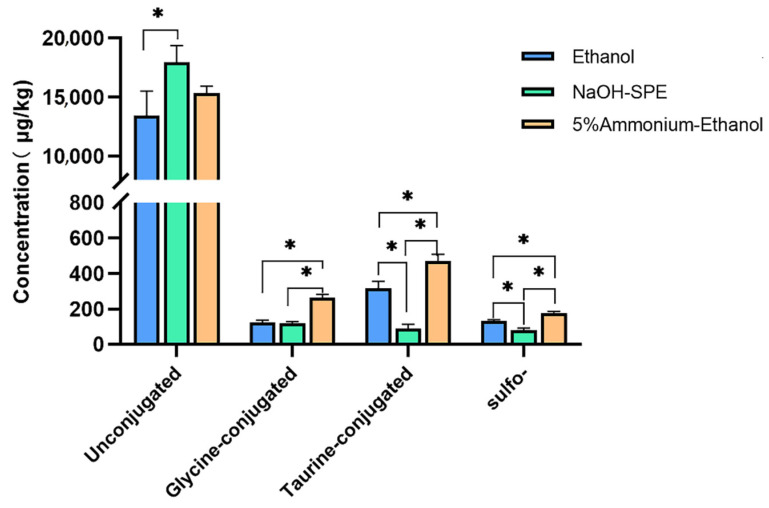
Extraction conditions comparison of bile acids profiles. Differences considered significant (*p* < 0.05) are denoted by *.

In order to develop a simple, time-saving, and high BA species coverage extraction method for different fecal biological samples, three different procedures were compared, including ethanol extraction (S1), reversed-phase SPE with high pH (S2), and high pH ethanol (S3) extraction. The pooled sample was used to evaluate the above protocols. As shown in Figure 1, S2 protocol NaOH-SPE gave the highest concentrations for unconjugated BAs but a much lower content of conjugated and sulfated BAs than the other two protocols (*p* < 0.05). In this protocol, one-hour preincubation for the sample before extraction may be responsible for this low conjugated content, especially the taurine-conjugated BAs. The dehydrogenase and desulfatase enzymes of the fecal microorganism may hydrolyze conjugated and sulfated BAs during this preincubation period [31]. Ethanol extraction (S1) also gave a lower yield for glycine and taurine conjugated BAs than the high pH ethanol protocol (S3). The alkaline condition could benefit from breaking the bonds between conjugated BA and fecal protein [32]. To summarize, the S3 protocol (5% ammonium–ethanol) was chosen in further experiments as a routine extraction protocol for feces.

### 2.2. Chromatography Separation Optimization

**Figure 2:**
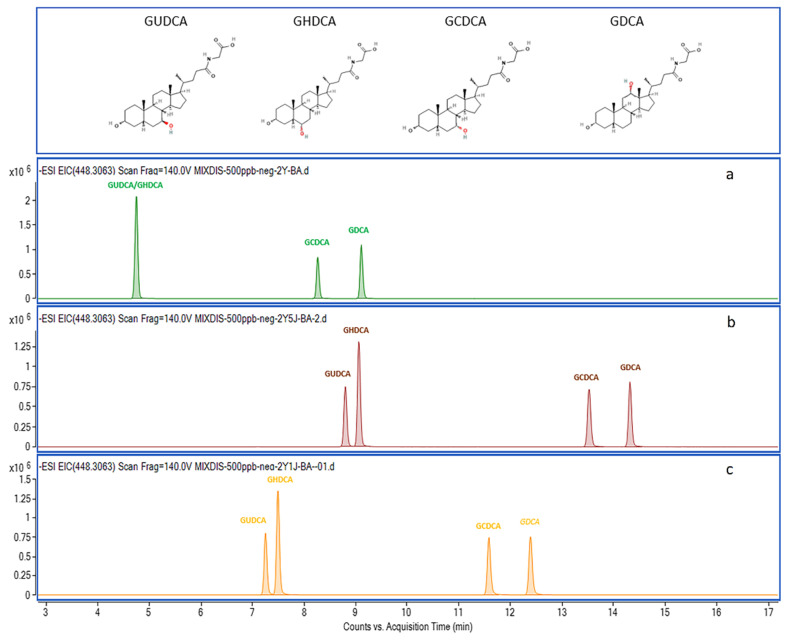
Mobile phase optimization for isobaric compounds chromatography separation. The GUDCA, GHDCA, GCDCA, and GDCA have the same molecular formula, C_26_H_43_NO_5_ ([M − H]− *m*/*z* 448.3063), but different -OH positions. (**a**) 2 mM ammonium acetate in aqueous solvent; (**b**) 2mM ammonium acetate and 0.05% formic acid in aqueous solvent; (**c**) 2 mM ammonium acetate and 0.01% formic acid in aqueous solvent.

**Figure 3:**
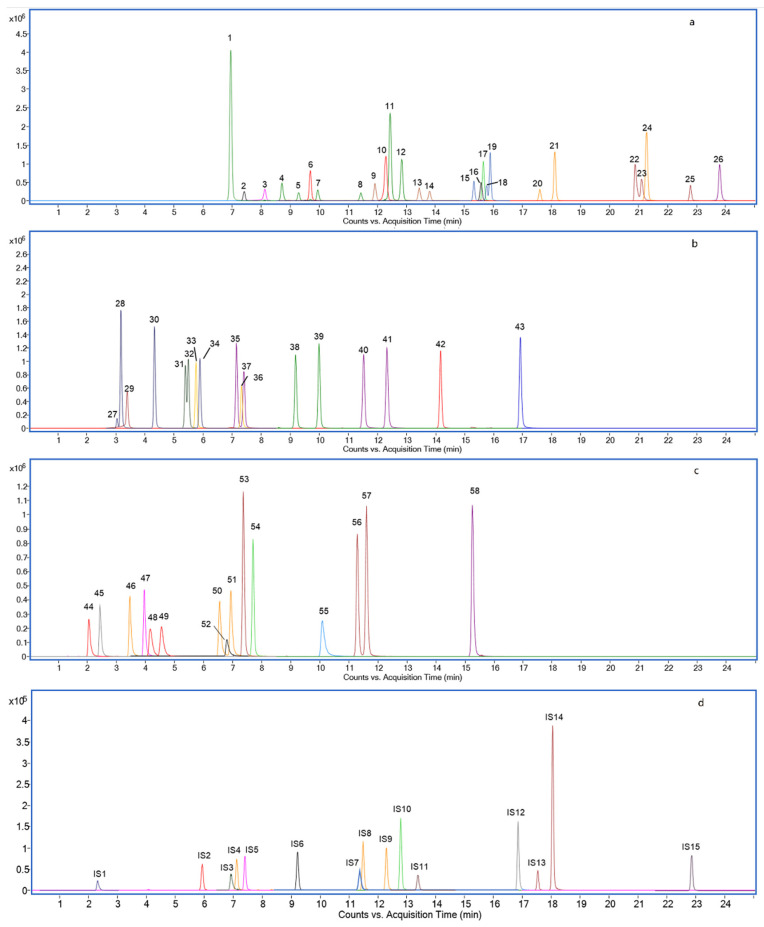
Extracted ion chromatograms of 26 unconjugated (**a**), 8 glycine-conjugated and 9 taurine-conjugated BAs (**b**), 5 taurine-sulfated, 5 glycine-sulfated, and 5 sulfated BAs (**c**), and 15 deuterium (D)-labeled BAs as Internal Standards (**d**). (1) UCA (2) 7,12-diketoLCA (3) DHCA (4) ω-MCA (5) α-MCA (6) 7-DHCA (7) β-MCA (8) γ-MCA (9) MDCA (10) 3-DHCA (11) alloCA (12) CA (13) UDCA (14) HDCA (15) 7-ketoLCA (16) 6,7-diketoLCA (17) norDCA (18) apoCA (19)12-ketoLCA (20) CDCA (21) DCA (22) isoalloLCA (23) isoDCA (24) isoLCA (25) LCA (26) 3-ketoLCA (27) T-α-MCA (28) T-β-MCA (29) GDHCA (30) THCA (31) TUDCA (32) THDCA (33) GHCA (34) TCA (35) GHDCA (36) GCA (37) GUDCA (38) TCDCA (39) TDCA (40) GDCA (41) GCDCA (42) TLCA (43) GLCA (44) TUDCA-3S (45) TCA-3S (46) GUDCA-3S (47) GCA-3S (48) TCDCA-3S (49) TDCA-3S (50) GCDCA-3S (51) GDCA-3S (52) TLCA-3S (53) UDCA-3S (54) CA-3S (55) GLCA-3S (56) CDCA-3S (57) DCA-3S (58) LCA-3S (IS1) TUDCA-3S-d4 (IS2) TCA-d4 (IS3) GDCA-3S-d4 (IS4) GUDCA-d4 (IS5) GCA-d4 (IS6) TCDCA-d5 (IS7) CDCA-3S-d4 (IS8) GDCA-d4 (IS9) GCDCA-d4 (IS10) CA-d4 (IS11) UDCA-d4 (IS12) GLCA-d4 (IS13) CDCA-d4 (IS14) DCA-d4 (IS15).

For high-resolution mass spectrometry, effective chromatography separation reduces matrix effects and improves the accuracy of identification and quantification. Therefore, the mobile phase constitution was optimized. Ammonium-based buffer and formic acid are common additives in aqueous solvents (mobile phase A) for negative ionization mode [33]. Additionally, the acidic condition was beneficial for the separation of BA structural isomer. For example, GUDCA, GHDCA, GCDCA, and GDCA have the same molecular formula, C_26_H_43_NO_5_, but different in -OH position (shown in Figure 2). The separations of their addition ion forms [M-H]^−^ (*m*/*z* 448.3063) were significantly affected by ammonium acetate and formic acid additive amount. GUDCA and GHDCA were not separated under individual ammonium acetate conditions (Figure 2a). When 0.05% formic acid was added in the mobile phase simultaneously, isobaric BAs can be differentiated (Figure 2b). Furthermore, the analysis times of these analytes could save 1–2 min when formic acid addiction volume was 0.01% instead of 0.05% (Figure 2c). Therefore, in terms of peak shapes, analysis times, and solvent saving, the best mobile phase compromise was 2 mM ammonium acetate and 0.01% formic acid in H_2_O. This is because weakly acidic mobile phase conditions contribute to the deprotonation of the analytes [34]. Information about the observed ions and retention times (RTs) of all analyzed compounds is given in Table 1. The extracted ion chromatograms (EICs) of all the unconjugated, glycine-conjugated, taurine-conjugated BAs, sulfated Bas, and deuterium-labeled BAs are shown in Figure 3.

### 2.3. Method Validation

The method was then validated following the recommendations for bioanalytical method validation [35] and confirmed to be selective and specific. For 58 BA analytes, the LOD ranged from 0.01 to 0.24 μg/kg (corresponding 0.15–3.26 nM) and the LOQ from 0.03 to 0.81 μg/kg (corresponding 0.44–9.77 nM) (Table S1). Our quantification limits were lower than previously reported values, 10–12.5 nM (methanol extraction in wet feces and detection by LC-MS/MS) [8] and 12.6–73.2 nM (NaOH-SPE extraction in dried feces and detection by LC-MS/MS) [32].

All calibration curves covered quantities from 0.5 to 1000 μg/L (corresponding 1–2000 nM) were linear, with squared correlation coefficients (r^2^) ranging from 0.995 to 0.999 without extra weighting analysis in the calibration curves algorithm. In other HRMS-based methods, nonlinear correlations have been observed in the quantitative analysis [23]. The possible reason is the limited dynamic range of the detector. Therefore, different regression algorithms (linear and quadratic with 1/x or 1/x^2^ weightings) were used in calibration curves calculation.

The accuracy of 58 bile acids at low, medium, and high spiked concentrations were 92.39–115.55%, 89.14–111.04%, and 94.88–106.25%, respectively. The precisions of intra-day and inter-day, expressed as variation coefficients %, were less than 10% (0.04–9.78% and 0.12–9.98%, respectively). Results are shown in Table S1.

The recovery percentages of three biological fecal matrices were evaluated at different concentration levels. Table S2 presented the recovery of BA in each biological sample. All the recovery values ranged from 80 to 120%.

The matrix effect percentages were evaluated at different concentration levels of each biological sample; detailed results are listed in Table S3. Matrix effect values were considered negligible, being in a range of 80–120% for all analytes in rat, mouse, and human fecal matrix samples. An appropriate extraction method, high-resolution detection instrument, and calibration of the internal standard could effectively reduce the matrix effect [24]. Moreover, the usage of 15 deuterium-labeled standards (1 for each of the 15 analyzed BA species) also compensated for the matrix effects. All these results were acceptable according to the FDA guidelines [35].

### 2.4. BA Profiling in Humans, Rats, and Mice

**Figure 4:**
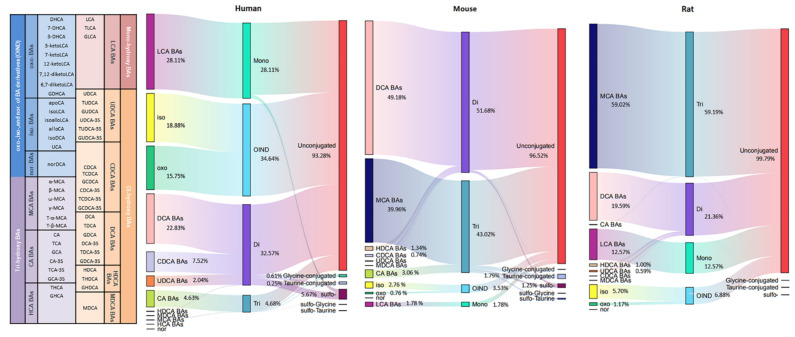
Sankey diagram of different bile acid concentration distribution in humans, rats, and mice.

The composition of individual BAs and the concentration proration of each BA in all their forms (unconjugated, amino acid conjugated (glycine or taurine), sulfo-conjugated, and double conjugated) are shown in Figure 4.

Unconjugated BAs were the most abundant BAs in humans, rats, and mice, all above 90%, with the corresponding BA indices of 93.28%, 96.52%, and 99.79%. Among the conjugated BAs, the highest sulfation of BAs was observed in humans (5.67%), while sulfation of BAs in mice and rats was 1.25% and 0.1%, respectively. Thakare et al. reported humans present better sulfation capability than rats and mice [28]. The higher percent sulfation capacity of humans is also consistent with plasma and urine matrix [25,36]. BA sulfation could increase BA hydrophilicity and promotes excretion in feces and urine, so it is an important mechanism for BA detoxification [30]. Glycine-conjugated BAs were predominant in humans, but only a tiny amount in rodent species, while the BA indices of taurine-conjugated were on the contrary. This tendency was consistent with previous reports [28]. Double conjugated BAs were also detected, but at very low levels, only 0.18% in humans, 0.41% in mice, and 0.03% in rats. For these double conjugated BAs, little data were reported in feces, which may be due to the limit of the extraction and the detection methods.

The number of hydroxyl groups affects the hydrophobic of BAs. The hydrophobicity was increased in the order of tri-OH BAs (CA, MCA, and HCA), di-OH BAs (CDCA and DCA), and mono-OH BA (LCA). The mono-OH BA (LCA) indices were more dominant in humans (28.11%) than in rodent species (mice 12.57% and rats 1.78%). The percentage of di-OH BAs was highest in rats (51.68%), followed by humans (32.57%), and was lowest in mice (21.36%). The percentage of tri-OH BAs was highest in mice (59.19%) and followed by rats (43.02%), and was lowest in humans (4.68%). Compared with humans, rodents possess a bigger hydrophilic BA pool because of the abundant presence of MCAs. It is well known that MCAs are scarcely reported in humans, but García-Cañaveras et al. and Li et al. detected MCAs in healthy and ESRD human serum, respectively [27,37]. Interestingly, for the first time, we detected ω-MCA and γ-MCA in ESRD patient fecal samples. The average concentration of total MCA was 0.7 μg/kg, and the percentage of these two MCA form BAs was 0.06%.

BAs have many derivatives, including iso-, oxo-, and nor- (OIND BAs). OIND BAs were highest in humans (34.64%), followed by mice (3.53%) and rats (6.88%). This result is opposite to the plasma, in which much higher OIND BAs were found in rats than in humans [25]. This might be due to the fact that more OIND BAs were absorbed by rats as compared to humans. Among these OIND BAs, the percentage of the form of iso- BAs was higher than oxo among human and rodent species (human iso- 18.88%, oxo- 15.75%, nor 0.01%; mouse iso- 2.76%, oxo- 0.76%, nor 0.01%; rat iso- 5.70%, oxo- 1.17%, nor 0.01%). However, higher oxo-BAs in plasma were reported [25]. It indicated that oxo-BA was easier to be reabsorbed into the blood, while iso-BA was excreted in feces.

### 2.5. BA Indices in Humans, Rats, and Mice

**Figure 5:**
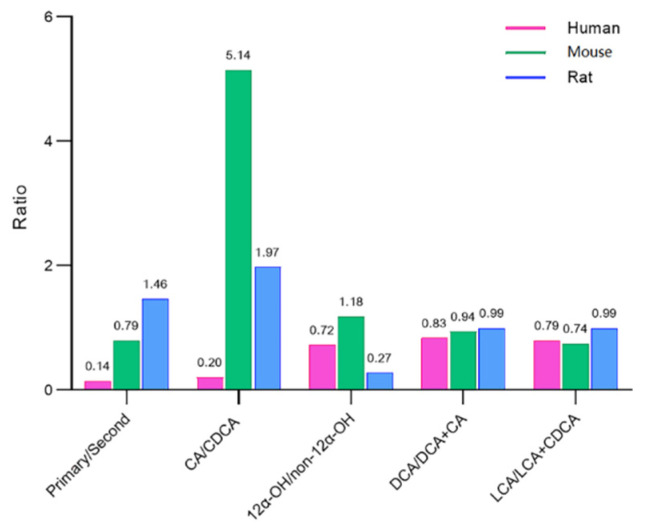
Bile acid indices in humans, rats, and mice.

The BA composition ratios are called BA indices, which could comprehensively assess the composition of BA pools, including primary to secondary BA ratio, CA/CDCA ratio, 12α-OH/non-12α-OH ratio, DCA/DCA+CA ratio, and LCA/LCA+CDCA ratio [28]. The BA indices are able to describe the metabolic transformation and biological function of BAs [25,28]. The BA indices vary among biological species, and the changes in BA indices could be an early warning of disease [29]. Therefore, in order to provide reference data for future research, the BA indices of human and rodent species (mice and rats) were present (Figure 5).

The ratio of primary to secondary BAs was calculated as the ratio of the sum of the concentrations of CDCA, CA, MCA, and HCA to the sum of the concentrations of DCA, LCA, UDCA, HDCA, and MDCA in all their forms [36]. This rate was lowest in humans (0.14), followed by mice (0.79) and rats (1.46). Rhishikesh et al. found the values of PBA/SBA in humans, rats, and mice were 1.1, 2.3, and 4.5 in plasma matrix while 0.8, 1.9, and 16.5 in urine matrix [28]. These results indicated that human gut microbiota is able to produce more abundant SBA than rodent species.

The ratio of total CA/total CDCA was quite lower in humans (0.20) than in rodents (mice 5.14 and rats 1.97). This ratio reflects the BA synthesis pathway preference (classical or alternative pathways) [36]. In the plasma and urine samples, this rate was also lower in humans than in mice and rats [28]. Thus, it could be speculated that BA synthesis is the preferred classical pathway in humans.

The ratio of 12α-OH/non-12α-OH was calculated as the ratio of the sum of the concentrations of DCA and CA to the sum of the concentrations of CDCA, HDCA, MDCA, LCA, UDCA, HCA, and MCA in all their forms. This ratio was highest in rats (1.18), followed by humans (0.72) and mice (0.27). This tendency was also in line with plasma and urine matrix. The extent of 12α-OH/non-12α-OH BA ratio has been linked to the deficiency of 12α-hydroxylase (CYP8B1) activity [38]. Moreover, several studies have reported that this ratio could reflect host metabolic status [2].

The 7α-dehydroxylase converts CA in DCA and CDCA in LCA, so the ratio of DCA/DCA+CA and the ratio of LCA/LCA+CDCA both reflected the 7α-dehydroxylase activity [9]. These two ratios were quite similar in human and rodent species, ranging from 0.74–0.99. Thus, it could be speculated that 7α-dehydroxylase activity was equal in these species.

## 3. Materials and Methods

### 3.1. Materials & Methods

LC-MS grade acetonitrile, ammonium acetate, and formic acid were obtained from Merck (Darmstadt, Germany). Water was obtained from the Merck Millipore Ultra-pure water purification system at 18.2 MΩ/cm (Merck Millipore, Darmstadt, Germany). SPE C18 columns (30 mg/300 cc) were purchased from Waters (Milford, CT, USA). The authentic compounds of 26 unconjugated, 8 glycine conjugated, 9 taurine conjugated, and 15 sulfo- BAs were ordered from either Toronto Research Chemicals (Toronto, ON, Canada), BePure (Bejing, China), or zzstandard^®^ (Shanghai, China). Fifteen deuterium D4-labeled BAs were purchased from BePure and were used as isotope-labeled internal standard (IS) for quantitation. The details on these authentic compounds are provided in Table 1.

### 3.2. Standard Solutions and Calibration Curves

Stock solutions (1 mg/mL each) of individual BAs were prepared by dissolving the respective compounds separately in methanol. These stock solutions were further diluted with methanol to give final concentrations of 0.01–50 mg/L. These standard solutions were used to determine the limits of detection (LODs) and the limits of quantitation (LOQs). A mixed-standard solution containing 100 μg/L of each of the 15 D4-labeled BAs was prepared in methanol and was used as the IS solution. For the preparation of the calibration curves, each working standard solution was mixed with an equal volume of the IS solution. The 58 standard stock solutions were then pooled together to obtain a 5 mg/L solution, further diluted in methanol to obtain 10 levels in the calibration curve ranging from 0.5–1000 μg/L.

### 3.3. Sample Preparation

Wet feces were thoroughly homogenized after reception and then stored at −80 °C. The fecal moisture content percentage of ESRD patients and rodent species were 70 ± 10% and 50 ± 5%, respectively. The sample was treated according to 3 extraction protocols (S1–S3).

#### 3.3.1. S1 Extraction with Ethanol

Briefly, 15 mg fecal sample was homogenized with 1 mL cold ethanol (containing Mix ISs) in 2 mL tubes filled with 3–4 mm glass beads. Homogenization was performed using an automated Precellys 24 Tissue Homogenizer (Bertin Technologies, Bretonneux, France) at middle speed for 30 s. The mixtures were centrifuged at 1350× *g* for 10 min at 4 °C. Supernatant was collected and dried under a nitrogen stream at 24 °C. The residue was dissolved in 150 μL initial mobile phase.

#### 3.3.2. S2 Extraction with 0.1 mol/L NaOH Followed by SPE

First, 1 mL of NaOH (0.1 mol/L) was added to 15 mg feces, Vortex shaken 30 s, and incubated for 1 h at 60 °C. Then, 2 mL water was added, homogenized 30 s, and centrifuged at 1350× *g* for 10 min at 4 °C. The supernatant was collected and purified with an SPE cartridge. The 30 mg SPE cartridge was pre-conditioned with 5 mL methanol and 5 mL water, then loaded with supernatant of extract solution and rinsed successively with 20 mL water, 10 mL hexane, and other 20 mL water. The BAs were then eluted with 5 mL methanol. The eluted fraction was collected and dried under a nitrogen stream at 24 °C. The residue was dissolved in a 150 μL initial mobile phase.

#### 3.3.3. S3 Extraction with 5% Ammonium-Ethanol

Briefly, 15 mg fecal sample was homogenized with 1 mL 5% Ammonium-Ethanol (contain Mix ISs) in 2 mL tubes filled with 3–4 mm glass beads. Homogenization was performed using an automated Precellys 24 Tissue Homogenizer (Bertin Technologies, Bretonneux, France) at middle speed for 30 s. The mixtures were centrifuged at 1350× *g* for 10 min at 4 °C. Another 1 mL extraction solution added, repeat the extraction process. Supernatant from the two extraction steps were pooled and dried under a nitrogen stream at 24 °C. The residue was dissolved in 150 μL initial mobile phase.

Reconstituted solution was diluted according to the endogenous BA content before LC injection. The results obtained from the analysis, expressed as μg/L of extract, were converted to μg/kg of dry feces by applying the following formula:

*C* = *C*_0_ × (*V*/*m*) × *n* × (1 − *M*_*F*_) (1)

where

*C* represents the concentration expressed as μg/kg;
*C*_0_ represents the concentration expressed as μg/L;
*V* represents the extraction volume (in L);
*m* represents the weight of wet feces (kg) subjected to extraction;
*n* represents the dilution ratio;
*M_F_* represents the fecal moisture content (%).

### 3.4. UPLC–Q-TOF Analysis

BAs analysis was performed by using an Agilent 1290 II UPLC system coupled with a G6545 quadrupole-time-of-flight mass spectrometer (UPLC–Q-TOF) from Agilent Technologies (Santa Clara, CA, USA), equipped with an Agilent Jet Stream electrospray (AJS ESI) source.

Separation of BAs was carried out using a using a BEH C18 (2.1 mm × 100 mm, 1.7 μm) UPLC column and a C18 guard column (2.1 × 10 mm, 1.7 µm), both from Waters Inc. (Milford, CT, USA). The column was kept at 30 °C. The mobile phase was consisted of 0.01% formic acid and 2 mM ammonium acetate in water (A) and acetonitrile (B). A linear gradient elution program was applied as follows: 0 min 25% B, 12.0 min 60% B, 26.0 min 75% B, 28.0 min 100% B, and hold on 2.0 min for equilibration. The flow rate was 0.3 mL/min, and the injection volume was 5 μL.

An Agilent Jet Stream electrospray ionization (ESI) interface was used, and its parameters were set as follows: dry gas temperature, 325 °C; dry gas flow, 7 L/min; nebulizer pressure, 35 psig; sheath gas temperature, 350 °C; and sheath gas flow 12 L/min, fragmentor voltage of 140 V and a capillary voltage of 3000 V. The detector operated in a low mass range (1700 *m/z*) and a 2 GHz extended dynamic range. In addition, the centroid mode was used for data collection and storage. Mass accuracy during the analysis was ensured by direct infusion into the source of a reference solution containing TFANH4 (112.9855 *m/z*) and HP-921 (1033.9881 *m/z*). This instrument gave a resolution greater than 10,000 full widths at half maximum (FWHM) at 112.985587 *m/z* and greater than 30,000 FWHM at 1633.949786 *m/z*. The analysis was carried out in the MS mode. The MS scan range was 100–1200 *m/z*, and the acquisition rate was two spectra per second. Data acquisition was accomplished using Agilent MassHunter Workstation Data Acquisition Version B.10.01 software (Santa Clara, CA, USA).

### 3.5. Standard Solutions and Calibration Curves

#### 3.5.1. Linearity, LOD, and LOQ

Linearity was determined by analysis of calibration curves for all commercially available standards of BAs. The method was validated using a ten-point calibration curve of 0.5–1000 μg/L.

The LOQ was defined as the lowest concentration at which the peak response was ten times that of the noise (10 S/N), and the LOD was the extrapolated concentration with a signal-to-noise ratio of three (3 S/N).

#### 3.5.2. Recovery and Matrix Effect

A relative blank matrix was used in method validation. In order to obtain the relative blank matrix, the fecal sample was extracted by extract solution, certificated, and vaporized solvent. The recovery and matrix effect were assessed at different spiked concentrations with six replicates. The spiked concentration of low, medium, and high were 2.5, 25, and 50 μg/kg, respectively. Moreover, according to the real endogenous BA content in different biological species, some higher spiked concentrations for individual abundant BA were also carried out, with detailed spiked concentrations shown in Table S1. The recovery was evaluated by comparing the peak areas of the analytes before extraction to the corresponding peak area in samples after extraction. The recovery rates must be within 100 ± 20%. The matrix effect was determined by comparing the peak area of post-extraction spiked BA and the standard solution of the same concentration.

#### 3.5.3. Precision and Accuracy

The precisions were evaluated as the intra- and inter-day coefficient of variation (CV, %) for BA analyses with a low spiked concentration in the pooled sample. The intra- and inter-day variations were determined using 5 replicates of spiked samples on the same day and on 5 different days. The precision CV was calculated from the ratio of the relative standard deviation to the mean of the measured analyte concentration. The accuracies were evaluated using solutions with spiked samples with low, medium, and high three certain concentrations, and the accuracy was calculated from the measured/theoretical concentrations×100%. The acceptable range of precision and accuracy for the maximal variation is within±20% according to the FDA guidelines [35].

#### 3.5.4. Clinical and Preclinical Samples Collection

This methodology application in ESRD patients was an ancillary study to our ESRD patient lipid metabolism trial. Therefore, twenty ESRD patients (10 males and 10 females, aged from 35 to 69) were randomly selected from total of 284 original samples. These patients underwent regular hemodialysis at the hemodialysis center in Beijing, China. Fresh fecal samples of patients were collected from all bowel motions before hemodialysis in hospital. Then individual samples were homogenized immediately and frozen at −80 °C.

Six C57BL/6 mice (male, 8 weeks of age) were purchased from the Experimental Animal Center of the First Affiliated Hospital of Tianjin University of Traditional Chinese Medicine. Six Sprague-Dawley (SD) rats (male, 8 weeks age) were purchased by Beijing HFK Bioscience Co. Ltd. (Beijing, China). Mice and rats were housed at animal facility with free access to a normal diet and water at 22 ± 2 °C, the relative humidity of 45 ± 15%, and a 12 h light/dark cycle. All experiments were approved by the Animal Ethical and Welfare Committee. The discharged feces of each rodent were collected into a centrifuge tube and frozen at −80 °C.

#### 3.5.5. Data Processing and Statistical Study

For LC-MS/MS data, MassHunter Quantitative Analysis vB.10.01 (Agilent Technologies, Inc., Santa Clara, CA, USA) was used for quantification. If the analyte concentration was below the LOQ, the value of LOQ/2 was used for statistical calculation. Tukey HSD all-pairwise comparisons test was used for multiple comparisons of data. A difference of *p* < 0.05 was considered significant. Statistical analysis and graphs were performed in GraphPad Prism v8.0.1 (GraphPad Software, San Diego, CA, USA). Sankey diagram was used in this study to visualize the BA types distribution in each biological species under the R Graph Gallery (https://www.data-to-viz.com/graph/sankey.html, accessed on 1 May 2022). Different BA types (classified by BA structure, detailed in Table 1) are represented by rectangles. Their links are represented with arcs that have a width proportional to the sub-category of the BA types.

## 4. Conclusions

In this study, we developed and validated a simple, effective and sensitive UPLC–Q-TOF method that simultaneously performs quantitative and qualitative analysis of 58 BAs, including unconjugated, amino acid conjugated (glycine or taurine), sulfo-conjugated, and double conjugated, as well as iso-, nor-, and oxo- BA metabolites in feces. All the methodology results were acceptable according to the FDA guidelines. This method could be applied in the global profiling of BA in humans, rats, and mice. In general, a higher proportion of sulfated BAs and mono-OH BA (LCA) was present in humans rather than in rodents. OIND BAs were also abundant in humans, especially iso- and oxo- BA. In terms of BA indices, PBA/SBA ratio and total CA/total CDCA ratio were fairly low in humans, while the DCA/DCA + CA ratio and LCA/LCA + CDCA ratio were equal in humans and rodents.

## Figures and Tables

**Table 1:** Bile acid analyte structural, quantitative, and qualitative information.

Compounds	Formula	RT ^1^	Transition	Adduct Ion	IS ^2^
Unconjugated					
Nordeoxycholic acid (norDCA)	C_23_H_38_O_4_	15.63	377.2697	[M − H]−	CA-d4
Dehydrocholic acid (DHCA)	C_24_H_34_O_5_	8.10	401.2333	[M − H]−	CA-d4
7,12-Diketolithocholic acid (7,12-diketoLCA)	C_24_H_36_O_5_	7.40	449.2545	[M + COOH]−	CA-d4
6,7-Diketolithocholic acid (6,7-diketoLCA)	C_24_H_36_O_5_	15.56	403.2490	[M − H]−	CA-d4
3-Ketolithocholic acid (3-ketoLCA)	C_24_H_38_O_3_	23.75	373.2748	[M − H]−	CA-d4
7-Ketolithocholic acid (7-ketoLCA)	C_24_H_38_O_4_	15.35	435.2752	[M + COOH]−	CA-d4
12-Ketolithocholic acid (12-ketoLCA)	C_24_H_38_O_4_	9.68	405.2646	[M − H]−	CA-d4
7-Ketodeoxycholic acid (7-DHCA)	C_24_H_38_O_5_	12.29	405.2646	[M − H]−	CA-d4
3-Ketodeoxycholic acid (3-DHCA)	C_24_H_38_O_5_	15.90	389.2679	[M + COOH]−	CA-d4
Apocholic acid (apoCA)	C_24_H_38_O_4_	15.75	435.2752	[M − H]−	CA-d4
Isolithocholic acid (isoLCA)	C_24_H_40_O_3_	21.23	375.2905	[M − H]−	CA-d4
Allolithocholic acid (isoalloLCA)	C_24_H_40_O_3_	21.00	375.2905	[M − H]−	CA-d4
Allocholic acid (alloCA)	C_24_H_40_O_5_	12.44	407.2803	[M − H]−	CA-d4
Ursocholic acid (UCA)	C_24_H_40_O_5_	6.92	407.2803	[M − H]−	CA-d4
Lithocholic acid (LCA)	C_24_H_40_O_3_	22.91	421.2959	[M + COOH]−	LCA-d4
Ursodeoxycholic acid (UDCA)	C_24_H_40_O_4_	13.41	437.2909	[M + COOH]−	UDCA-d4
Chenodeoxycholic acid (CDCA)	C_24_H_40_O_4_	17.58	437.2909	[M + COOH]−	CDCA-d4
Isodeoxycholic acid (isoDCA)	C_24_H_40_O_4_	21.25	391.2854	[M − H]−	DCA-d4
murideoxycholic acid (MDCA)	C_24_H_40_O_4_	11.85	437.2909	[M + COOH]−	CA-d4
Deoxycholic acid (DCA)	C_24_H_40_O_4_	18.10	391.2854	[M − H]−	CA-d4
Hyodeoxycholic acid (HDCA)	C_24_H_40_O_4_	13.78	437.2909	[M + COOH]−	CA-d4
α-Muricholic acid (α-MCA)	C_24_H_40_O_5_	9.39	453.2860	[M + COOH]−	CA-d4
β-Muricholic acid (β-MCA)	C_24_H_40_O_5_	10.04	453.2858	[M + COOH]−	CA-d4
γ-Muricholic acid (γ-MCA)	C_24_H_40_O_5_	11.41	453.2858	[M + COOH]−	CA-d4
ω-muricholic acid (ω-MCA)	C_24_H_40_O_5_	8.70	453.2858	[M + COOH]−	CA-d4
Cholic acid (CA)	C_24_H_40_O_5_	12.83	407.2803	[M − H]−	CA-d4
Glycine-conjugated					
Glycodehydrocholic acid (GDHCA)	C_26_H_37_NO_6_	3.34	458.2548	[M − H]−	GCA-d4
Glycolithocholic acid (GLCA)	C_26_H_43_NO_4_	16.81	432.3119	[M − H]−	GLCA-d4
Glycodeoxycholic acid (GDCA)	C_26_H_43_NO_5_	12.25	448.3063	[M − H]−	GDCA-d4
Glycochenodeoxycholic acid (GCDCA)	C_26_H_43_NO_5_	11.44	448.3063	[M − H]−	GCDCA-d4
Glycoursodeoxycholic acid (GUDCA)	C_26_H_43_NO_5_	7.09	448.3068	[M − H]−	GUDCA-d4
Glycohyocholic acid (GHCA)	C_26_H_43_NO_6_	5.78	464.3018	[M − H]−	GCA-d4
Glycohyodeoxycholic acid (GHDCA)	C_26_H_43_NO_5_	7.34	448.3068	[M − H]−	GCA-d4
Glycocholic acid (GCA)	C_26_H_43_NO_6_	7.36	464.3018	[M − H]−	GCA-d4
Taurine-conjugated					
Taurolithocholic acid (TLCA)	C_26_H_45_NO_5_S	14.24	482.2946	[M − H]−	TCA-d4
Taurochenodeoxycholic acid (TCDCA)	C_26_H_45_NO_6_S	9.23	498.2895	[M − H]−	TCDCA-d5
Taurodeoxycholic acid (TDCA)	C_26_H_45_NO_6_S	10.05	498.2895	[M − H]−	TCDCA-d5
Tauroursodeoxycholic acid (TUDCA)	C_26_H_45_NO_6_S	5.48	498.2895	[M − H]−	TCDCA-d5
Taurohyodeoxycholic acid (THDCA)	C_26_H_45_NO_6_S	5.58	498.2895	[M − H]−	TCDCA-d5
Tauro α-Muricholic acid (T-α-MCA)	C_26_H_45_NO_7_S	3.19	514.2844	[M − H]−	TCA-d4
Tauro β-Muricholic acid (T-β-MCA)	C_26_H_45_NO_7_S	4.34	514.2844	[M − H]−	TCA-d4
Taurohyocholic Acid (THCA)	C_26_H_45_NO_7_S	5.92	514.2844	[M − H]−	TCA-d4
Taurocholic acid (TCA)	C_26_H_45_NO_7_S	3.04	514.2844	[M − H]−	TCA-d4
Sulfo-					
Lithocholic Acid-3-Sulfate (LCA-3S)	C_24_H_40_O_6_S	15.27	455.2473	[M − H]−	CDCA-3S-d4
Ursodeocycholic Acid-3-Sulfate (UDCA-3S)	C_24_H_40_O_7_S	7.40	471.2422	[M − H]−	CDCA-3S-d4
Chenodeoxycholic Acid-3-Sulfate (CDCA-3S)	C_24_H_40_O_7_S	11.32	471.2422	[M − H]−	CDCA-3S-d4
Deoxycholic Acid-3-Sulfate (DCA-3S)	C_24_H_40_O_7_S	11.63	471.2422	[M − H]−	CDCA-3S-d4
Cholic Acid-3-Sulfate (CA-3S)	C_24_H_40_O_8_S	7.72	487.2371	[M − H]−	CDCA-3S-d4
Glycine-Sulfo-					
Glycolithocholic Acid-3-Sulfate (GLCA-3S)	C_26_H_43_NO_7_S	9.98	512.2687	[M − H]−	GDCA-3S-d4
Glycoursodeoxycholic Acid-3-Sulfate (GUDCA-3S)	C_26_H_43_NO_8_S	3.42	528.2637	[M − H]−	GDCA-3S-d4
Glycochenodeoxychlolic Acid-3-Sulfate (GCDCA-3S)	C_26_H_43_NO_8_S	6.50	528.2637	[M − H]−	GDCA-3S-d4
Glycodeoxycholic Acid-3-Sulfate (GDCA-3S)	C_26_H_43_NO_8_S	6.90	528.2637	[M − H]−	GDCA-3S-d4
Glycocholic Acid-3-Sulfate (GCA-3S)	C_26_H_43_NO_9_S	3.91	544.2586	[M − H]−	GDCA-3S-d4
Taurine-Sulfo-					
Taurolithocholic Acid-3-Sulfate (TLCA-3S)	C_26_H_45_NO_8_S_2_	6.98	562.2514	[M − H]−	TUDCA-3S-d4
Tauroursodeoxycholic Acid-3-Sulfate (TUDCA-3S)	C_26_H_45_NO_9_S_2_	2.06	578.2463	[M − H]−	TUDCA-3S-d4
Taurochenodeoxycholic Acid-3-Sulfate (TCDCA-3S)	C_26_H_45_NO_9_S_2_	4.19	578.2463	[M − H]−	TUDCA-3S-d4
Taurodeoxycholic Acid-3-Sulfate (TDCA-3S)	C_26_H_45_NO_9_S_2_	4.57	578.2463	[M − H]−	TUDCA-3S-d4
Taurocholic Acid-3-Sulfate (TCA-3S)	C_26_H_45_NO_10_S_2_	2.46	594.2412	[M − H]−	TUDCA-3S-d4
Internal standard					
Cholic acid-d4 (CA-d4)	C_24_H_36_D_4_O_5_	12.86	411.3054	[M − H]−	
Lithocholic Acid-d4 (LCA-d4)	C_24_H_36_D_4_O_3_	22.96	379.3156	[M − H]−	
Ursodeoxycholic Acid-d4 (UDCA-d4)	C_24_H_36_D_4_O_4_	13.45	395.3105	[M − H]−	
Chenodeoxycholic Acid-d4 (CDCA-d4)	C_24_H_36_D_4_O_4_	17.54	395.3105	[M − H]−	
Deoxycholic Acid-d4 (DCA-d4)	C_24_H_36_D_4_O_4_	18.13	395.3105	[M − H]−	
Glycolithocholic Acid-d4 (GLCA-d4)	C_26_H_39_D_4_NO_4_	16.86	436.3370	[M − H]−	
Glycoursodeoxycholic Acid-d4 (GUDCA-d4)	C_26_H_39_D_4_NO_5_	7.12	452.3320	[M − H]−	
Glycodeoxycholic Acid-d4 (GDCA-d4)	C_26_H_39_D_4_NO_5_	6.90	452.3320	[M − H]−	
Glycochenodeoxycholic Acid-d4 (GCDCA-d4)	C_26_H_39_D_4_NO_5_	12.26	452.3320	[M − H]−	
Glycocholic Acid-d4 (GCA-d4)	C_26_H_39_D_4_NO_6_	7.43	468.3269	[M − H]−	
Taurocholic Acid-d4 (TCA-d4)	C_26_H_41_D_4_NO_7_S	5.96	518.3095	[M − H]−	
Taurochenodeoxycholic Acid-d5 (TCDCA-d5)	C_26_H_40_D_5_NO_6_S	9.26	503.3209	[M − H]−	
Chenodeoxycholic Acid-3-Suflate-d4 (CDCA-3S-d4)	C_24_H_36_D_4_O_7_S	11.38	475.2673	[M − H]−	
Glycodeoxycholic Acid-3-Sulfate-d4 (GDCA-3S-d4)	C_26_H_39_D_4_NO_8_S	11.42	532.2888	[M − H]−	
Tauroursodeoxycholic Acid-3-Sulfate-d4 (TUDCA-3S-d4)	C_26_H_41_D_4_NO_9_S_2_	2.25	582.2714	[M − H]−	

^1^ RT—retention time. ^2^ IS:—internal standard.

## Data Availability

The data presented in this study are available in the article and Supplementary Materials.

## Supplementary Materials

The following supporting information can be downloaded at: https://www.mdpi.com/article/10.3390/metabo12070633/s1, Table S1: Sensitivity, linearity, accuracy, and precision of bile acids by ultra-high-performance liquid chromatography coupled with quadrupole-time-of-flight mass spectrometer analysis; Table S2: The recovery (%) of spiked concentration (μg/kg) for bile acids in each biological sample; Table S3: The matrix effect (%) of spiked concentration (μg/kg) for bile acids in each biological sample.
