# Supplementary material for: Quantitative Profiling of Bile Acids in Feces of Humans and Rodents by Ultra-High-Performance Liquid Chromatography–Quadrupole Time-of-Flight Mass Spectrometry
Source: Metabolites. 2022 Jul 11;12(7):633. doi: 10.3390/metabo12070633 (PMC9323729; doi:10.3390/metabo12070633)
Supplement: Supplementary file 1 [file metabolites-12-00633-s001.zip › metabolites-1771551-supplementary.pdf]

Table S1. Sensitivity, linearity, accuracy, and precision of bile acids by ultra-high-performance liquid chromatography coupled with quadrupole-time-of-flight mass spectrometer analysis.

Compounds	Linear Formula	LOD [1] (μg/kg)	LOQ [2] (μg/kg)	Precision (%)		Accuracy (%)		
				CV[3] Intra	CV Inter	Low	Medium	High
norDCA	y = 0.651386 x + 8.381732E-004	0.03	0.09	1.19	2.88	102.43	100.37	97.93
DHCA	y = 0.450615 x - 7.223210E-005	0.06	0.19	5.66	1.60	97.65	100.81	100.18
7,12-diketoLCA	y = 0.331379 x + 2.739396E-004	0.08	0.27	0.42	2.17	101.59	102.81	98.81
6,7-diketoLCA	y = 0.502384 x + 6.877313E-004	0.08	0.28	0.19	5.00	98.05	101.30	99.61
3-ketoLCA	y = 0.323258 x - 0.004134	0.15	0.50	1.54	0.21	99.60	100.38	100.83
7-ketoLCA	y = 1.533500 x + 0.003542	0.03	0.11	0.77	8.37	104.91	101.85	98.66
12-ketoLCA	y = 0.499284 x + 2.390257E-004	0.02	0.12	1.17	2.37	103.55	98.55	101.05
7-DHCA	y = 0.388852 x - 0.001835	0.03	0.24	2.96	0.19	103.51	99.64	99.57
3-DHCA	y = 0.662617 x + 0.001249	0.07	0.08	4.51	4.10	94.89	111.04	101.55
apoCA	y = 0.277331 x + 1.573897E-004	0.09	0.30	8.10	1.09	115.55	106.22	106.25
isoLCA	y = 0.784308 x + 0.001987	0.05	0.18	8.87	6.42	93.08	101.21	100.82
isoalloLCA	y = 1.182057 x + 7.166959E-004	0.03	0.10	1.51	3.94	99.60	100.75	100.87
alloCA	y = 3.528376 x + 6.996823E-004	0.02	0.06	1.60	0.92	99.17	95.71	97.69
UCA	y = 6.058174 x + 0.003005	0.01	0.03	3.04	6.81	99.09	97.29	102.48
LCA	y = 1.009131 x + 0.030949	0.02	0.05	2.67	0.24	100.53	102.29	98.02
UDCA	y = 1.734014 x + 0.001762	0.05	0.16	1.78	5.20	105.58	101.29	100.08
CDCA	y = 1.412708 x + 9.372573E-004	0.04	0.12	6.71	3.73	98.59	96.06	100.39
isoDCA	y = 1.226037 x + 0.001227	0.02	0.07	6.23	5.15	101.08	100.86	99.49
MDCA	y = 0.837269 x + 2.348623E-004	0.03	0.11	0.97	1.86	103.05	100.81	99.76
DCA	y = 0.778721 x + 0.007314	0.02	0.08	4.22	7.39	97.86	102.05	100.93
HDCA	y = 0.465608 x - 3.247209E-004	0.06	0.21	4.45	5.48	107.44	98.12	95.47
α-MCA	y = 1.191828 x + 0.001271	0.04	0.13	5.23	3.81	101.62	102.24	102.53
β-MCA	y = 1.216027 x + 0.002118	0.03	0.10	4.21	8.35	107.83	105.21	103.69
γ -MCA	y = 1.401082 x + 7.317450E-004	0.03	0.10	0.17	6.19	103.39	100.25	99.87
ω-MCA	y = 1.470009 x + 0.023447	0.02	0.08	8.70	5.28	98.13	102.35	101.86
CA	y = 1.457358 x + 0.006402	0.02	0.08	9.78	0.79	106.04	103.18	105.02
GDHCA	y = 0.658480 x - 0.013658	0.04	0.14	5.70	1.76	105.33	96.20	98.54
GLCA	y = 1.120251 x - 0.004391	0.03	0.09	0.40	7.26	105.12	98.30	99.81
GDCA	y = 1.211294 x + 0.001554	0.04	0.14	3.65	2.47	99.37	102.33	98.36
GCDCA	y = 1.229572 x + 5.999672E-004	0.03	0.11	4.10	0.12	97.94	101.00	102.82
GUDCA	y = 1.561018 x - 0.015192	0.04	0.14	7.29	7.83	103.11	100.25	98.59
GHCA	y = 1.134934 x - 0.036098	0.04	0.10	0.04	5.29	100.99	104.23	100.11
GHDCA	y = 0.825538 x + 5.799325E-004	0.03	0.14	6.34	2.07	106.42	95.12	97.70
GCA	y = 0.795128 x + 0.006782	0.03	0.11	7.42	2.27	102.50	96.89	103.62
TLCA	y = 1.702910 x + 0.001291	0.03	0.10	6.94	0.34	100.89	102.46	98.92

TCDCA	y = 1.156731 x + 0.004892	0.03	0.10	7.74	3.98	103.56	102.80	99.13
TDCA	y = 1.831847 x + 5.272285E-004	0.03	0.09	4.52	7.77	109.69	106.03	99.15
TUDCA	y = 1.079787 x + 0.006024	0.03	0.11	8.77	0.29	102.36	103.64	100.50
THDCA	y = 1.353724 x - 8.004187E-004	0.04	0.12	9.05	4.74	96.30	100.09	98.42
T-β-MCA	y = 1.844287 x + 0.003743	0.03	0.11	2.52	4.30	106.81	101.75	94.90
THCA	y = 1.788719 x - 0.009246	0.04	0.12	1.46	2.24	100.32	99.87	102.19
TCA	y = 1.396524 x + 7.956564E-004	0.06	0.19	5.40	8.22	100.53	99.69	99.69
T-α-MCA	y = 0.165350 x - 0.002958	0.14	0.47	8.10	0.52	99.87	100.42	98.87
LCA-3S	y = 1.498371 x + 0.013112	0.04	0.13	1.70	3.54	101.84	101.87	96.22
UDCA-3S	y = 1.576279 x + 0.004861	0.03	0.10	8.42	7.30	106.03	99.74	94.89
CDCA-3S	y = 1.249296 x + 0.004990	0.03	0.11	5.17	9.98	104.81	100.90	94.88
DCA-3S	y = 1.493935 x + 0.020683	0.03	0.09	1.46	4.57	105.67	102.07	96.71
CA-3S	y = 1.087670 x + 0.012822	0.05	0.18	5.61	4.66	101.67	103.65	96.09
GLCA-3S	y = 1.009221 x - 0.031716	0.13	0.43	0.56	3.43	105.02	97.16	98.80
GUDCA-3S	y = 0.998073 x - 0.007271	0.09	0.31	0.94	5.98	99.75	97.86	103.65
GCDCA-3S	y = 1.023463 x - 0.009598	0.10	0.33	3.83	5.28	96.68	90.53	102.19
GDCA-3S	y = 1.126820 x - 0.007818	0.08	0.27	1.43	4.36	104.50	89.14	104.63
GCA-3S	y = 1.032762 x - 0.003273	0.09	0.30	3.50	3.41	103.65	96.96	102.96
TLCA-3S	y = 1.319584 x - 0.004526	0.24	0.81	3.77	6.93	92.39	98.99	102.89
TUDCA-3S	y = 1.179070 x + 0.013669	0.08	0.27	3.62	8.73	101.91	103.05	99.66
TCDCA-3S	y = 1.213847 x + 0.003391	0.11	0.38	6.33	6.90	106.13	101.90	99.74
TDCA-3S	y = 1.267394 x + 0.007063	0.11	0.38	6.64	5.21	105.06	98.60	100.89
TCA-3S	y = 1.470925 x - 0.003398	0.08	0.26	2.36	1.60	100.81	102.26	98.44

1 LOD—limits of detection
2 LOQ—limits of quantification
3 CV—coefficient variations

Table S2. The recovery (%) of spiked concentration (μg/kg) for bile acids in each biological sample.

Compounds	Rat							Mouse							Human						
	2.5	25	50	250	500	2500	5000	2.5	25	50	250	500	2500	5000	2.5	25	50	250	500	2500	5000
norDCA	108.93	98.95	103.89					95.62	98.32	108.42					117.38	99.75	114.25				
DHCA	106.45	93.30	104.49					108.60	95.66	107.40					104.09	101.03	109.96				
7,12-diketoLCA	105.32	98.94	104.44					80.22	96.39	113.56					115.34	117.31	113.22				
6,7-diketoLCA	107.60	100.34	99.31					111.32	104.95	108.73					91.53	96.54	93.37				
3-ketoLCA	109.49	95.25	111.95	96.99	105.44			105.09	120.83	92.17	102.69	102.72			103.36	93.20	103.96	99.50	99.34		
7-ketoLCA	108.67	100.99	103.38					101.14	95.60	106.35					91.37	94.95	92.28	107.10	90.19	102.69	
12-ketoLCA	96.16	94.99	104.87					84.15	97.43	108.32					108.09	88.94	98.27	101.24	85.19	105.99	
7-DHCA	102.83	101.38	98.57	112.46	103.06	118.65	112.15	106.73	98.78	103.67	100.72	98.19	113.58	105.47	100.08	96.83	102.04	117.78	103.25	93.49	
3-DHCA	83.91	93.46	102.33	106.02	104.21	116.63	112.08	117.78	97.35	107.59	99.19	100.21	118.65	101.61	110.44	81.12	81.47	119.34	88.96	108.54	
apoCA	82.78	103.70	83.56					103.47	112.19	95.53					89.11	101.45	116.95				
isoLCA	118.96	118.09	103.78					107.27	116.27	114.47					106.11	96.94	90.73				
isoalloLCA	105.75	100.52	92.81					115.82	97.12	99.27					102.83	105.38	97.98				
alloCA	85.43	99.16	85.91	114.53	91.63			92.20	108.41	111.19	101.31	99.29			96.62	101.55	105.96	107.96	101.40	97.92	99.76
UCA	89.89	106.97	105.43					118.79	99.31	111.07					80.32	97.13	88.24	112.51	103.96	105.46	95.74
LCA	102.16	101.33	102.93					111.59	100.05	108.75					120.54	110.19	94.90	92.65	91.33	107.08	98.76
UDCA	104.86	98.88	102.52					97.47	101.07	107.68					100.86	94.35	113.38				
CDCA	104.47	96.25	104.05					103.76	98.98	110.07					109.18	98.64	114.80				
isoDCA	95.85	89.39	106.39	100.65	110.58	116.20	108.58	103.53	115.65	114.97	103.85	98.21	115.11	103.60	117.01	103.51	113.38	109.79	93.17	103.99	98.86
MDCA	100.51	100.29	103.08	113.84	104.90			118.66	110.12	108.81	101.08	100.48			100.26	95.51	117.93				
DCA	84.71	97.77	107.68	115.66	107.24	119.46	112.41	114.08	107.18	107.97	102.41	105.82	117.89	105.66	102.85	103.08	112.28				
HDCA	106.98	98.06	105.57	109.30	110.95	117.13	110.58	92.94	109.84	114.59	103.77	103.68	112.66	106.18	103.35	103.97	115.97				
α-MCA	100.38	89.59	105.87	111.06	105.85			101.93	94.75	108.09	100.22	99.70			106.84	106.76	102.24				
β-MCA	107.78	100.14	102.90					98.65	99.66	110.74					83.10	96.29	113.67				
γ -MCA	106.06	94.96	102.35	110.70	108.10	112.54	114.09	116.00	99.54	109.22	101.76	102.66	120.66	112.06	111.24	88.71	81.04				
ω-MCA	87.55	82.30	88.17	110.97	110.55	119.39	111.53	103.17	96.74	120.81	115.12	107.02	114.09	104.28	83.89	82.72	88.64				
CA	89.27	87.11	104.60	106.38	104.31	116.47	110.75	114.11	99.06	113.95	98.84	102.08	114.93	104.97	95.46	101.39	109.69	107.12	92.82	111.46	93.19
GDHCA	99.10	98.85	101.05					98.52	96.94	104.17					117.01	94.26	115.08				
GLCA	116.43	92.61	100.06					106.31	100.19	106.21					103.52	113.01	112.42				
GDCA	103.43	95.95	104.67					100.55	100.95	108.50					95.07	82.07	107.51				
GCDCA	96.98	99.61	105.70					96.08	106.42	101.87					92.38	82.80	101.08				

GUDCA	104.59	104.01	100.31			91.86	102.93	107.10			99.92	89.11	112.78			
GHCA	116.61	112.44	101.24			91.01	92.10	103.58			83.57	96.55	99.38			
GHDCA	92.70	100.80	95.95			99.55	101.06	101.70			91.17	101.62	95.60			
GCA	112.35	96.92	100.23			112.10	95.55	105.24			101.02	80.22	97.25			
TLCA	104.41	99.80	105.69			107.70	106.15	108.33			93.36	94.86	96.45			
TCDCA	84.92	79.48	106.24	111.38	109.27	108.71	98.76	104.64	99.13	99.94	90.01	94.47	117.19			
TDCA	103.01	96.07	103.01			100.95	98.56	109.96			118.39	93.51	109.90			
TUDCA	94.05	99.52	105.89	113.31	109.21	93.23	97.49	110.76	98.39	103.61	99.61	94.21	110.88			
THDCA	80.99	97.25	98.44			106.09	103.96	113.75			103.00	100.26	90.43			
T-β-MCA	104.58	104.04	104.98			108.01	99.18	100.66			114.29	94.37	106.81			
THCA	108.89	100.01	101.60	106.16	102.87	110.06	98.16	107.81	96.89	101.00	113.68	100.16	105.41			
TCA	98.79	84.76	101.35	113.31	108.69	115.81	102.32	101.97	100.23	101.30	108.38	108.91	103.64			
T-α-MCA	109.73	92.74	101.63	113.40	105.93	111.59	111.65	108.61	95.11	104.64	93.19	100.69	102.31			
LCA-3S	107.02	100.85	102.12			116.46	98.19	109.57			100.72	99.32	107.15	105.06	99.94	96.84
UDCA-3S	105.72	102.82	101.10			109.37	98.22	105.57			105.42	103.83	102.86	113.08	94.47	97.95
CDCA-3S	91.62	82.47	106.11			104.91	100.64	106.20			104.74	97.23	105.98	99.76	98.56	106.68
DCA-3S	100.58	107.63	103.78			102.95	103.27	110.31			102.45	100.71	99.22	96.52	95.40	96.22
CA-3S	103.55	100.24	101.82			102.98	99.45	110.11			95.63	93.15	101.66	101.01	98.22	93.07
GLCA-3S	103.87	101.66	100.61			119.15	95.38	105.20			99.28	107.88	107.87			
GUDCA-3S	131.94	101.37	98.71			93.86	106.90	101.82			91.49	100.12	102.43			
GCDCA-3S	126.66	87.54	101.78			118.40	85.21	106.49			95.41	96.92	99.84			
GDCA-3S	110.11	105.98	102.97			101.12	80.47	111.00			109.73	108.99	98.78			
GCA-3S	100.63	93.83	100.55			98.16	98.22	110.66			99.69	98.49	98.69			
TLCA-3S	94.28	96.48	107.01			116.77	94.55	109.38			95.96	99.83	97.12			
TUDCA-3S	103.54	107.48	105.78			82.02	100.25	110.41			99.54	96.67	96.96			
TCDCA-3S	100.54	102.85	117.10			111.10	97.64	116.98			102.43	102.52	105.02			
TDCA-3S	105.13	97.12	105.54			106.01	102.31	106.75			90.88	106.23	95.52			
TCA-3S	82.46	80.50	104.88	100.00	104.34	94.02	100.17	108.18	98.95	96.16	101.88	100.33	94.27			

Table S3. The matrix effect (%) of spiked concentration (μg/kg) for bile acids in each biological sample.

Compounds	Rat							Mouse							Human						
	2.5	25	50	250	500	2500	5000	2.5	25	50	250	500	2500	5000	2.5	25	50	250	500	2500	5000
norDCA	103.64	103.78	105.84					100.12	100.86	104.21					106.60	110.34	104.33				
DHCA	93.94	87.11	116.66					98.96	88.91	108.14					122.12	102.26	106.37				
7,12-diketoLCA	91.32	97.97	106.96					99.26	102.07	103.04					88.31	104.22	92.00				
6,7-diketoLCA	96.27	103.03	103.95					101.99	99.52	104.57					112.40	109.81	117.02				
3-ketoLCA	106.04	91.73	96.96	98.09	87.49			102.79	100.89	64.23	103.54	98.76			110.22	108.57	108.20	95.58	111.27		
7-ketoLCA	103.66	99.17	103.12					109.42	98.78	106.04					111.81	84.51	111.48	95.17	109.48	101.08	
12-ketoLCA	81.31	104.61	105.21					83.71	86.09	103.14					90.35	110.12	97.09	98.64	108.33	99.20	
7-DHCA	111.20	100.23	108.85	98.61	106.30	99.73	98.99	100.67	108.65	111.19	105.45	110.95	108.98	106.87	114.66	99.65	81.16	113.40	109.02	98.46	
3-DHCA	112.18	102.51	110.86	100.06	103.92	103.71	97.70	103.07	108.02	109.01	103.23	105.76	119.53	106.34	98.70	103.06	100.45	84.13	100.74	102.93	
apoCA	80.05	84.93	88.55					118.15	106.44	82.78					101.69	87.51	85.88				
isoLCA	97.35	101.62	88.08					86.69	90.56	94.17					93.35	96.92	108.08				
isoalloLCA	110.62	91.47	116.74					102.35	121.56	123.14					88.52	107.91	99.12				
alloCA	85.47	89.60	97.52	93.66	107.70			136.48	110.46	104.35	104.56	103.56			88.90	97.59	98.54	87.14	112.83	103.46	99.76
UCA	112.65	93.69	105.91					108.59	110.97	103.22					106.70	94.33	117.13	85.47	105.51	100.77	95.74
LCA	111.51	99.79	103.52					116.20	106.94	100.95					93.68	103.20	85.70	82.84	84.05	99.24	98.76
UDCA	98.71	103.13	103.27					98.62	93.56	105.01					120.16	110.22	99.87				
CDCA	104.70	104.60	104.78					108.56	94.32	106.75					118.74	98.06	94.76				
isoDCA	86.67	80.52	111.21	98.59	106.61	103.49	98.39	85.98	108.14	107.53	97.45	99.05	104.67	102.45	96.17	98.63	86.95	88.74	112.58	100.71	98.86
MDCA	100.84	107.69	104.87	98.03	104.76			104.66	108.00	102.76	87.54	90.54			102.82	99.89	93.85				
DCA	102.28	109.14	107.70	94.23	108.38	102.31	98.48	93.76	106.63	105.57	98.65	103.56	98.67	102.46	120.91	106.77	99.30				
HDCA	108.15	118.81	119.66	106.74	113.54	103.35	99.01	114.90	106.89	91.68	109.54	96.55	98.03	104.62	96.13	102.93	107.84				
α-MCA	113.32	99.28	103.60	95.84	106.95			103.37	107.73	102.33	98.34	90.35			81.99	97.85	96.93				
β-MCA	101.87	98.80	103.73					106.37	104.64	103.22					105.16	97.27	84.06				
γ -MCA	98.74	105.25	102.81	97.95	107.11	108.19	94.92	95.26	105.42	104.67	109.45	114.32	116.34	98.54	97.58	93.40	95.86				
ω-MCA	115.54	100.05	103.05	96.43	104.70	101.58	97.45	80.52	106.21	97.96	115.12	107.02	114.09	104.28	86.78	94.58	99.42				
CA	82.90	94.05	100.53	95.16	111.00	100.85	98.06	96.34	108.79	100.02	99.34	105.34	116.32	102.54	100.67	96.07	86.83	84.34	95.94	95.00	93.19
GDHCA	118.86	100.97	110.45					120.45	114.76	111.14					93.98	114.29	101.06				
GLCA	94.11	105.61	101.21					97.74	103.76	101.34					103.61	99.15	98.53				
GDCA	106.22	104.28	106.27					109.05	108.68	105.12					98.31	104.81	95.33				

GCDCA	113.80	104.66	107.38			113.70	109.54	106.67			106.92	84.38	95.62			
GUDCA	101.15	101.65	99.01			107.85	109.76	104.21			113.83	115.14	100.32			
GHCA	103.57	89.27	107.23			104.83	108.97	110.87			102.84	106.82	98.79			
GHDCA	100.44	100.36	104.76			116.52	112.23	113.14			95.36	84.64	94.25			
GCA	100.69	98.77	99.62			102.52	104.56	106.23			111.09	108.15	96.92			
TLCA	112.53	104.50	103.15			119.88	110.67	98.95			80.47	108.44	97.01			
TCDCA	102.38	99.12	103.38	95.96	103.87	110.74	105.12	106.35	90.32	93.21	118.54	107.28	96.36			
TDCA	98.54	101.32	104.38			109.18	106.76	105.07			85.80	106.00	97.19			
TUDCA	106.83	99.23	101.01	94.41	107.80	115.95	106.31	99.85	109.32	112.45	96.42	105.63	97.93			
THDCA	85.03	106.05	102.47			90.54	97.56	98.41			97.25	95.06	101.89			
T-β-MCA	118.82	99.16	100.10			103.39	99.05	105.76			103.37	106.70	112.66			
THCA	102.35	100.63	105.38	98.28	113.40	100.29	113.01	104.82	90.34	87.40	94.65	112.25	96.44			
TCA	93.15	101.76	105.94	96.46	103.30	101.77	105.81	108.59	98.03	104.34	87.46	92.82	87.62			
T-α-MCA	85.51	105.71	103.22	91.50	107.00	105.98	106.89	105.19	98.45	113.23	93.16	102.62	97.05			
LCA-3S	103.60	99.50	103.02			104.27	98.67	102.96			90.52	81.51	91.30	82.15	109.05	106.98
UDCA-3S	98.72	100.63	101.78			91.20	100.53	103.56			96.40	111.78	114.95	103.41	111.58	106.15
CDCA-3S	94.91	98.95	108.90			107.70	103.24	107.32			93.90	81.48	83.07	93.99	108.52	92.73
DCA-3S	103.57	99.37	102.65			95.27	116.56	105.66			83.30	95.92	99.28	101.33	108.28	104.62
CA-3S	108.69	101.88	97.03			108.23	107.68	98.81			114.26	97.42	99.91	96.65	108.77	102.69
GLCA-3S	113.67	99.43	99.10			88.49	104.64	99.84			94.24	110.79	100.70			
GUDCA-3S	85.62	102.96	105.81			102.24	103.56	111.77			93.02	107.99	100.14			
GCDCA-3S	94.39	97.12	108.16			105.45	98.65	113.25			101.22	119.14	117.18			
GDCA-3S	103.49	81.38	106.18			112.74	102.67	107.66			102.10	103.09	106.40			
GCA-3S	106.23	101.88	107.32			101.94	102.45	110.02			110.93	87.51	100.63			
TLCA-3S	105.10	100.97	112.20			94.09	98.75	110.07			97.21	101.55	115.57			
TUDCA-3S	107.09	98.31	106.81			108.58	97.53	103.99			90.34	105.28	107.20			
TCDCA-3S	106.44	92.27	95.13			98.76	97.42	91.30			102.26	119.02	112.44			
TDCA-3S	111.60	107.33	104.44			86.47	114.52	106.93			96.88	91.18	94.10			
TCA-3S	92.96	98.99	110.03	99.64	103.26	105.66	107.82	103.74	98.95	96.16	102.03	89.90	99.75			
